# Systematisches Review zur Effektivität von Lokalanästhetika bei der Therapie von neuropathischen Schmerzen oder Phantomschmerzen

**DOI:** 10.1007/s00101-025-01500-1

**Published:** 2025-02-24

**Authors:** Joanna Kastelik, Karsten Schwerdtfeger, Annette Stolle, Michael Schäfer, Sascha Tafelski

**Affiliations:** 1 Klinik für Anästhesiologie und Intensivmedizin, Campus Charité Mitte, Charité – Universitätsmedizin Berlin, corporate member of Freie Universität Berlin and Humboldt-Universität zu Berlin, Charitéplatz 1, 10117 Berlin, Deutschland; 2 Schmerzmedizin Campus Charité Mitte, Klinik für Anästhesiologie und Intensivmedizin, Charité – Universitätsmedizin Berlin, Charitéplatz 1, 10117 Berlin, Deutschland; 3 Klinik für Neurochirurgie, Medizinische Fakultät der Universität des Saarlandes, 66421 Homburg (Saar), Deutschland; 4 Andreas Wentzensen Forschungsinstitut, BG Klinik Ludwigshafen, Ludwig-Guttmann-Str. 13, 67071 Ludwigshafen, Deutschland

**Keywords:** Lokalanästhesie, Nervenverletzungen, Chronischer Schmerz, Neuroplastizität, Literaturanalyse, Local anaesthesia, Nerve injury, Chronic pain, Neuroplasticity, Literature analysis

## Abstract

**Hintergrund:**

Im September 2023 wurde die überarbeitete S3-Leitlinie *Versorgung peripherer Nervenverletzungen *publiziert. Multimodale schmerztherapeutische Behandlungsstrategien haben hierbei Eingang in die Leitlinie gefunden und schließen systemische und lokal-medikamentöse, physiotherapeutische und ergotherapeutische Maßnahmen mit ein. Eine zentrale Fragestellung bewertete dabei die viel diskutierte Behandlungsoption mittels perineuraler Lokalanästhesie.

**Ziel der Arbeit:**

Um die Effektivität lokaler Infiltrationen bei der Behandlung von neuropathischen Schmerzen nach einer Nervenverletzung darzustellen, erfolgen eine systematische Literaturrecherche und Evidenzbewertung mittels Metaanalyse.

**Material und Methoden:**

Nach Formulierung einer entsprechenden PICO(„patient/population, intervention, comparison and outcomes“)-Frage (Infobox 1) innerhalb der Leitliniengruppe erfolgte eine selektive Literaturanalyse zu klinischen Studien in Datenbanken (PubMed, Cochrane Central Register of Controlled Trials – CENTRAL) bis zum 31.07.2023. Zwei Reviewer bewerteten die Literatur und prüften systematische Reviews auf zusätzliche Literaturverweise.

**Ergebnisse:**

Insgesamt wurden 357 Publikationen identifiziert. Nach Entfernung von Duplikaten (*n* = 15) wurden *n* = 327 Publikationen bewertet. In der vertiefenden Literaturanalyse wurde schlussendlich eine relevante Studie identifiziert und in die Evidenzbewertung eingeschlossen.

**Diskussion:**

Lokalanästhetikainfiltrationen stellen eine Therapieoption von Neuropathien nach Amputationen dar. Ein RCT (Randomisierte kontrollierte Studie) zeigte über 4 Wochen nach mehrtägiger perineuraler Lokalanästhetikainfiltration eine Reduktion von Schmerzen und schmerzbedingter Funktionseinschränkung. Weitere Studien sind erforderlich, um einen höheren Evidenzgrad zur Effektivität dieser Therapieform ableiten zu können.

**Graphic abstract:**

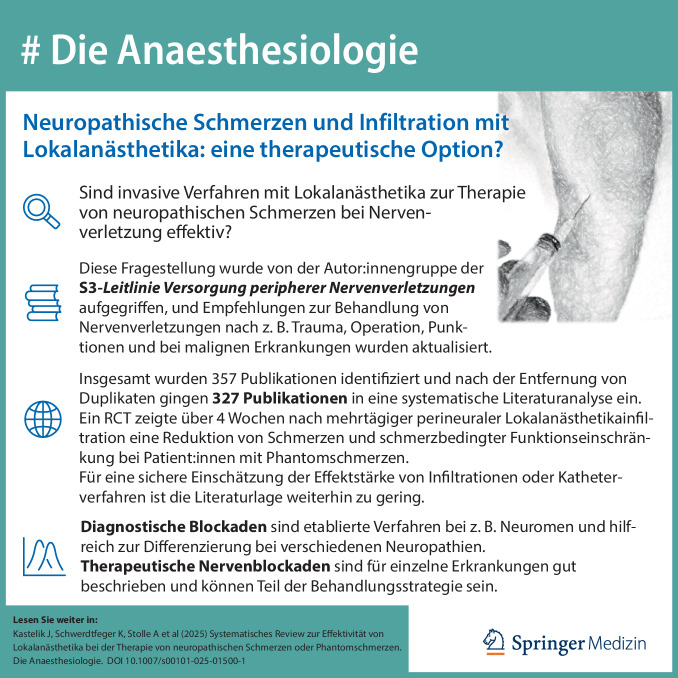

## Abstract (en)

**Background:**

Chronic pain is still a relevant medical and socioeconomic problem. The treatment focuses not only on pain reduction but also on functional treatment goals. Neuropathic pain includes biological, social and psychological aspects. In September 2023, the updated S3 guidelines for the management of peripheral nerve injuries were published. Multimodal pain management strategies encompassing systemic and local pharmacological, physiotherapeutic and occupational therapeutic interventions, are part of the guidelines. A central question addressed the widely debated treatment option using perineural local anaesthetics.

**Objective:**

The aim of the study was to evaluate the effectiveness of local anaesthetic infiltration in the treatment of neuropathic pain following nerve injuries through a systematic literature review and evaluation of the evidence by a meta-analysis.

**Material and methods:**

After formulating a PICO (patient/population, intervention, comparison and outcomes) question (Infobox 1) within the guideline group, a selective literature analysis of controlled trials in databases (PubMed, Cochrane Central Register of Controlled Trials—CENTRAL) was conducted until 31 July 2023. The literature was assessed by two reviewers and systematic reviews were examined for additional references. The studies were assessed using the Risk of Bias Tool 2.0 of the Cochrane Collaboration for randomized trials and the evidence was classified according to the GRADE (Grading of Recommendations, Assessment, Development and Evaluation) system.

**Results:**

A total of 357 publications were identified in the literature search. After removing duplicates (*n* = 15) 327 publications were evaluated. The literature analysis showed heterogeneity with respect to the pain localization, local anaesthetics and reported outcomes. In an in-depth literature analysis one relevant study was identified and included in the evaluation of the evidence. This study enrolled and randomized 144 patients between December 2013 and October 2018 and evaluated the effectivity of the continuous infusion of local anaesthetics (lidocaine 2% with epinephrine 2.5 µg/ml as an initial bolus in both study groups followed by an infusion of ropivacaine 0.5% in the intervention group over 6 days) on the intensity of the phantom pain in comparison to the placebo group with a continuous infusion of saline over 6 days. The mean pain intensity and pain-related dysfunctions were reduced in the intervention group after 4 weeks. In the intervention group 25 patients and in the placebo group 40 patients received the crossover treatment after 4 weeks.

**Conclusion:**

Infiltration with local anaesthetics represents a potential therapeutic option for neuropathic pain and phantom pain after amputations. A randomized, blinded, placebo-controlled study from 2021 demonstrated lower pain intensity and a reduction in pain-related functional limitations after 4 weeks of continuous perineural local anesthetic infiltration. Further studies are necessary to establish a higher level of evidence regarding the effectiveness of minimally invasive pain treatment with local anaesthetics. In particular, long-term follow-up is necessary to be able to draw conclusions with respect to the analgesic efficacy of infiltration with local anaesthetics.

## Hintergrund

Im September 2023 wurde die S3-Leitlinie *Versorgung peripherer Nervenverletzungen* unter Mitbeteiligung der Deutschen Schmerzgesellschaft überarbeitet [9]. Chronische Schmerzen und insbesondere neuropathische Schmerzen stellen ein relevantes medizinisches und sozioökonomisches Problem dar [25]. Für die Schmerztherapie sind Nervenverletzungen nach Traumata, Operationen, Virusinfektionen oder durch maligne Erkrankungen ein bedeutendes Feld. Pathophysiologisch entstehen periphere Nervenverletzungen dabei durch äußere Noxen wie Schnitte, mechanische oder virale Einwirkungen sowie iatrogen durch physikalische (z. B. Elektrotrauma, Kälte, Strahlung) oder chemischen Läsionen (z. B. Injektionen). Nicht zuletzt kann sich nach einer Nervenverletzung ein komplexes regionales Schmerzsyndrom (CRPS) vom Typ II entwickeln oder ein Neurom als spezifische neuronale Komplikation manifestieren. Nicht wenige Patient:innen sind insofern aufgrund von Nervenverletzungen auf eine längere Anbindung an schmerztherapeutische Einrichtungen angewiesen oder werden durch neurologische oder neurochirurgische Behandler:innen versorgt. Neuropathische, chronische Schmerzen beinhalten verschiedene biologische, soziale und psychologische Dimensionen [2]. Die S3-Leitlinie legt vor diesem Hintergrund einen wichtigen Schwerpunkt auf die Diagnostik und verschiedene Therapieoptionen nach Nervenverletzungen [9]. Die invasive Therapie mittels Lokalanästhetika hat eine lange medizinische Tradition [15]. Erste Berichte der Behandlung von Neuralgien mit Lokalanästhetikainjektionen lassen sich bis ins Jahr 1892 zurückverfolgen [1]. So haben Lokalanästhetika seit dem 20. Jh., lokal appliziert als auch perineural als Leitungsanästhesie, eine zunehmende Anwendung erfahren [29]. Demgegenüber entwickelte sich eine kritische Evaluation dieser Therapieverfahren unter Berücksichtigung von nachhaltigen Behandlungseffekten. Interventionelle Verfahren beinhalten ausgeprägte inhärente Placebowirkungen [23], die zu einer eher zurückhaltenden Indikationsstellung dieser Verfahren in der konservativen Schmerztherapie geführt haben [34]. Im Rahmen der Überarbeitung der S3-Leitlinie ergab sich dabei die Notwendigkeit einer aktuellen Literaturbewertung zu Fragestellung, inwiefern lokale Infiltrationen mittels Katheterverfahren oder Infiltrationsserien bei neuropathischen Schmerzen oder Phantomschmerzen zu einer Reduktion der Schmerzintensität führen könnten. Ausgehend von dieser Fragestellung wurde eine eigene PICO(„patient/population, intervention, comparison and outcomes“)-Frage formuliert und einer systematischen Literaturanalyse zugeführt. Insgesamt wurden 7 PICO-Fragen formuliert, die im Rahmen der Evidenzanalyse im Leitlinienprozess bearbeitet werden sollten. Diese Fragestellungen lassen sich im Methodenreport der Leitlinie abrufen [9].

Gegenstand dieser Arbeit ist es, über die erfolgte Literaturanalyse zu berichten und die aktuelle Evidenz zur Behandlung von spezifischen Neuropathien bzw. Phantomschmerzen mit lokalanästhesiologischen Katheterverfahren oder Infiltrationsserien als minimalinvasive Therapieoptionen zu evaluieren.

## Material und Methoden

Die Expertenkommission der S3-Leitlinie *Versorgung peripherer Nervenverletzungen* formulierte im Rahmen des aktuell publizierten Updates der Leitlinie [9] die folgende PICO-Frage:

- **„Patients“**: Patient:innen mit neuropathischen Schmerzen oder Phantomschmerzen
- **„Intervention“**: lokale Infiltration mittels Katheterverfahren oder Infiltrationsserien mittels Lokalanästhetika
- **„Control“**: Placebo oder Standardbehandlung
- **„Outcomes“**: Schmerzreduktion (visuelle Analogskala, VAS), Phantomschmerzinzidenz

## Studiendesign und Untersuchungsmethoden

Es wurde zunächst ein systematisches Review entsprechend den Kriterien der *Preferred reporting items for systematic reviews and meta-analyses* (PRISMA) [27] durchgeführt. Als **Studientyp** wurden prospektive und retrospektive kontrollierte klinische Studien sowie randomisierte kontrollierte Studien mit einem „cross over“ oder parallelen Design mit aktiven oder placebokontrollierten Studiengruppen berücksichtigt.

Als **Studienpopulation **waren Patient:innen mit neuropathischen Schmerzen oder Phantomschmerzen avisiert, da in einer ersten Literatursichtung deutlich wurde, dass spezifischere Indikationen wie Neurome oder Nervenverletzungen in der Literatur keine relevante Abbildung erfahren und geeignete Publikationen die IASP(„International Association for the Study of Pain“)-Nomenklatur *Neuropathische Schmerzen* verwendeten. Als **Intervention **wurde nach Infiltrationsserien mit Lokalanästhetika oder mittels katheterbasierter, kontinuierlicher Infusion von Lokalanästhetika gesucht. Als **Studien-Outcome **wurde die Schmerzintensität, die in der Literatur in der Regel als numerische Analogskala berichtet wird, gewählt. Reviews wurden untersucht, wenn sie das Outcome der Effektivität berücksichtigt haben, um zusätzliche Literatur über Querverweise identifizieren zu können [8, 18, 30]. Die **Literaturrecherche **erfolgte mittels der **PubMed**-Datenbank sowie des Cochrane Central Register of Controlled Trials (CENTRAL) bis zum 31.07.2023. Hierzu wurde ein Suchstring entwickelt, um die Trefferhäufigkeit mittels *MeSH („Medical Subject Headings“) terms* zu erhöhen. Tierexperimentelle Studien wurden a priori ausgeschlossen, als Sprache wurde nach englischen, deutschen oder französischen Publikationen gefiltert. Auf eine primäre Altersbeschränkung wurde dagegen verzichtet. Zwei Publikationen wurden zusätzlich über die Leitliniengruppe identifiziert.

## Auswahl von Studien

Nach Identifikation von relevanten Studien wurden zunächst Duplikate entfernt. Die resultierenden Publikationen wurden zunächst einem Screening von Titel und Abstracts zugeführt. Hierzu führten 2 Reviewer (S.T., K.S.) unabhängig voneinander eine Literaturanalyse durch. Bei fehlendem Konsens zu Studien wurden diese Untersuchungen in die nächste Analysephase überführt. In einer zweiten Stufe wurden die ausgewählten Publikationen einem Volltextscreening zugeführt. Die Bewertung der Studien erfolgte unabhängig durch 2 Reviewer (S.T., K.S.); bei fehlendem Konsens wurde die Literaturanalyse in der Expertengruppe der Leitlinie diskutiert. Die resultierende Publikationsliste wurde dann einer Evidenzbewertung zugeführt.

## Bewertung der methodischen Qualität/Datenanalyse

Eingeschlossene Studien wurden zur Einstufung des Verzerrungsrisikos mittels des Risk of Bias Tool 2.0 for randomized trials (RoB) der Cochrane Collaboration untersucht [22]. Dabei wurde das Verzerrungspotenzial anhand von 5 Domänen (Randomisierungsprozess, Abweichungen von den vorgesehenen Interventionen, fehlende Ergebnisdaten, Ergebnismessung, Selektion der berichteten Ergebnisse) beurteilt. Die Evidenzgraduierung erfolgte nach GRADE (Grading of Recommendations, Assessment, Development and Evaluation) entsprechend der Leitlinienmethodik der AWMF [9]. Zur Bewertung der Daten erfolgte eine ergänzende grafische Analyse (Forest-Plot der Effektstärke) [11, 31].

## Ergebnisse

**Abb. 1:**
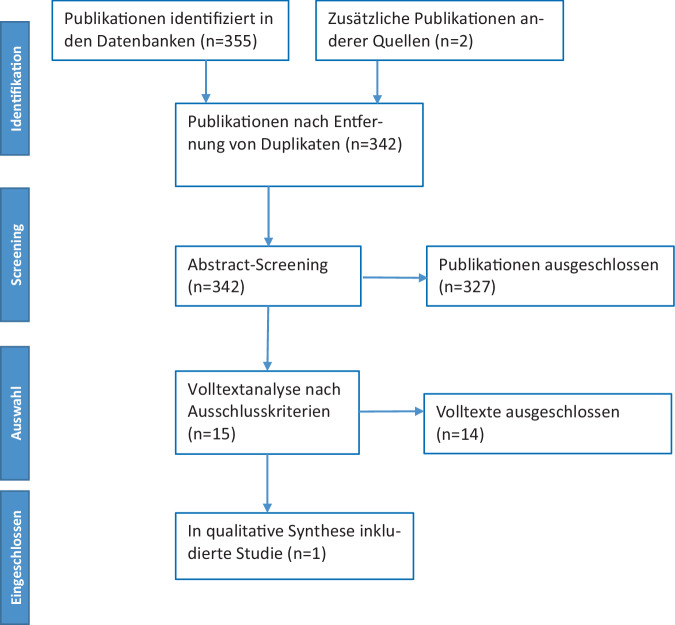
Einschlussdiagramm. (Nach der S3-Leitlinie Versorgung peripherer Nervenverletzungen [9])

**Abb. 2:**
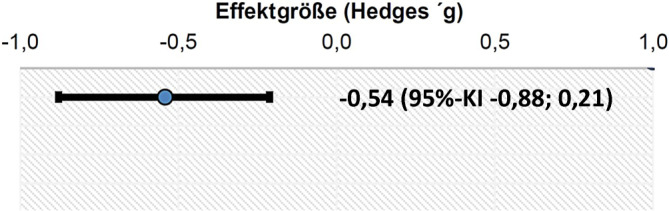
Forest-Plot der Effektgröße zur mittleren Phantomschmerzreduktion im Vergleich zwischen Interventionsarm und Placebogruppe aus Ilfeld et al. 2021 [21]. (Dargestellt ist die Effektgröße der Studie (Hedges’g))

Insgesamt wurden in der primären Literaturanalyse 357 Publikationen identifiziert und 15 Duplikate entfernt (Abb. 1). Weitere 327 Publikationen wurden nach dem primären Screening von Titel und Abstract als unpassend ausgeschlossen. Es resultierten insgesamt 15 Studien, die einer Volltextanalyse zugeführt wurden [3, 6–8, 14, 16–18, 21, 26, 28, 30, 32, 35, 36]. Die resultierende Literatur zeigte dabei eine große Heterogenität zu Indikationen, verwendeten Lokalanästhetika sowie Lokalisationen und v. a. hinsichtlich der berichteten Endpunkte. Insgesamt waren schmerztherapeutisch relevante, klinische Endpunkte mit einer zumindest mittelfristigen Nachverfolgung von Tagen oder Wochen nach der Intervention sehr selten; vielfach wurden kurzfristige Wirkungen von Lokalanästhetika aus mechanistischen Überlegungen heraus untersucht. Für die Beantwortung der PICO-Frage wurde schlussendlich eine Studie [21] bei Patient:innen mit manifestem Phantomschmerz eingeschlossen. Diese Studie von Ilfeld et al. [21] untersuchte den Einfluss einer kontinuierlichen perineuralen Infusion mit einem Lokalanästhetikum (Lidocain 2 % mit Epinephrin 2,5 µg/ml als Initialbolus in beiden Studiengruppen, anschließend Infusion von Ropivacain 0,5 % in der Interventionsgruppe) über 6 Tage auf die Intensität von Phantomschmerzen im Vergleich zu Placebo mit der Infusion von NaCl-Lösung. Die Untersuchung wurde als multizentrische, randomisierte, vierfach verblindete, placebokontrollierte Studie im Parallelgruppendesign im Studienzeitraum 2013 bis 2018 durchgeführt, und 144 Patient:innen wurden eingeschlossen (Tab. 1). Bis nach 4 Wochen zeigte sich in der Interventionsgruppe eine niedrigere Schmerzintensität im Vergleich zur Kontrollgruppe: die mittlere Phantomschmerzintensität wurde durch Lokalanästhetika um 2,4 (Standardabweichung ±3,0) Punkte und durch Placebo um 0,9 (Standardabweichung ±2,3) Punkte reduziert. Die Differenz zwischen den Gruppen betrug demnach im Mittel 1,5 Punkte mit einem 95 %-Konfidenzintervall von 0,5–2,4. Die Effektgröße der Phantomschmerzreduktion ist im Forest-Plot in Abb. 2 dargestellt. Die Studie berichtet zudem über positive Veränderung in schmerzbedingten Dysfunktionen als sekundäre Endpunkte durch die Intervention. Für die Patient:innen war nach 4 Wochen ein Cross over innerhalb der nächsten 12 Wochen möglich; 25 Patient:innen in der Interventionsgruppe und 40 Patient:innen in der Kontrollgruppe nahmen hieran teil.

**Tab. 1:** Effektivität der Schmerzreduktion zur Therapie von neuropathischen Schmerzen (Population: Patient:innen mit neuropathischem Schmerzen oder Phantomschmerzen; Intervention: lokale Infiltration mittels Katheterverfahren oder Infiltrationsserien mittels Lokalanästhetika; Vergleichsintervention: Placebo oder Standardbehandlung)

Endpunkt	Ergebnisse und Messwerte	Absolute Effektschätzer		Gewissheit der Evidenz (Vertrauenswürdigkeit der Evidenz)	Zusammenfassung
Zeitrahmen		Placebo oder Standard	Lokale Infiltration		
Phantomschmerz, NRS absolut	Gemessen mit: NRS-Skala: 0–10 – niedriger ist besser Basierend auf Daten von 144 Patienten und einer Studie [21]	*4,5* Mittelwert	*3,0* Mittelwert	*Moderat* Aufgrund von schwerwiegender unzureichender Präzision^1^	Die Studie von Ilfeld et al. 2021 zeigt 4 Wochen nach kontinuierlicher perineuraler Infusion mit einem Lokalanästhetikum über 6 Tage eine Abnahme des absoluten Phantomschmerzes im Vergleich zur Placebogruppe. Der Beobachtungszeitraum erscheint recht kurz.
Beobachtungszeit 4 Wochen		Differenz: *MD 1,5 kleiner * (95 %-KI 2,76 kleiner – 0 kleiner)			
Phantomschmerz, NRS Delta	Gemessen mit: NRS-Skala: 0–10 niedriger ist besser Basierend auf Daten von 144 Patienten und einer Studie [21]	*−0,9* Mittelwert	*−2,4* Mittelwert	*Moderat* Aufgrund von schwerwiegender unzureichender Präzision^1^	Die Studie von Ilfeld et al. 2021 zeigt 4 Wochen nach kontinuierlicher perineuraler Infusion mit einem Lokalanästhetikum über 6 Tage eine ausgeprägtere Abnahme des Phantomschmerzes im Vergleich zur Placebogruppe. Der Beobachtungszeitraum erscheint recht kurz.
Beobachtungszeit 4 Wochen		Differenz: *MD 1,5 kleiner * (95 %-KI 2,68 kleiner – 0 kleiner)			

^1^ *Unzureichende Präzision: schwerwiegend. *Daten von *n* = 1 Studie [21]

## Bewertung von Bias

Aufgrund des Cross-over-Designs der Studie können Endpunkte länger als 4 Wochen nicht ohne potenziellen Selektionsbias betrachtet werden. Die Autor:innen der Studie [21] weisen zudem auf den Aspekt der unklaren Dauer des analgetischen Effektes der perineuralen Infusionen und der optimalen Infusionsparameter hin. Die Qualität der Verblindung wurde in der Studie berichtet und zeigte sich durch die Nervenblockade über mehrere Tage als teilweise eingeschränkt. Insbesondere in der Kontrollgruppe war die Gruppenzuordnung durch Teilnehmende (50 %) eher richtig vermutet worden als in der Verumgruppe (38 %). Insgesamt ergibt sich für diese Studie ein moderates Risiko für Verzerrung (Tab. 2).

**Tab. 2:** Risiko für Bias (AWMF nach Cochrane Risk of Bias Tool 2.0 – RoB) in Ilfeld et al. [21]

Randomisierungsprozess	Niedrig
Abweichungen von den vorgesehenen Interventionen	Niedrig
Fehlende Ergebnisdaten	Niedrig
Ergebnismessung	Niedrig
Selektion des berichteten Ergebnisses	Moderat
*Insgesamt*	Moderat

## Quantitative Synthese

Aufgrund der Heterogenität der betrachteten Studien bestand keine Datenbasis für eine Metaanalyse von Daten. Insbesondere zeigte sich in der Literaturanalyse, dass neben der geringen Stichprobengröße und einem ungeeigneten Studiendesign auch nur wenige Studien über eine relevante Zeitspanne untersuchten. Zudem wäre für die spezifische Fragestellung der Phantomschmerzprävention wichtig, in Studien differenziert zwischen postoperativem Schmerz im Amputationsareal (Stumpfschmerz), einer spezifischen Neuropathie (Beispiel: Neurom) und einem Phantomschmerz zu berichten. Tab. 3 berichtet über identifizierte Studien, die nicht in eine quantitative Synthese eingeschlossen werden konnten.

**Tab. 3:** Weitere identifizierte Publikationen zur Lokalanästhetikaapplikation ohne quantifizierbare Endpunkte für eine Metaanalyse

Studie	Methodik und Patientenkollektiv	Ergebnis
Clendenen et al. [7]	Retrospektive Fallserie, chronischer postoperativer Schmerz nach Knieendoprothese, *n* = 16, Lokalanästhesie mit Bupivacain (Mercain + Steroid) und konsekutive Radiofrequenzablation des Ramus infrapatellaris	Ansprechrate 9/16 (56 %)
Casale et al. [6]	Randomisierte kontrollierte Pilotstudie, Patienten mit Phantomschmerz, *n* = 8, Cross-over-Design. Lokalanästhetikaapplikation *kontralateral* zur Amputation (Bupivacain 0,25 % vs. NaCl 0,9 %)	Nach 1 h höhere Schmerzreduktion unter Lokalanästhetika (−5,3) als unter Placebo (−1,5). Aufgrund des Cross-over-Designs keine längeren Daten
Boelens et al. [4]	Randomisierte kontrollierte Studie zum „anterior cutaneous nerve entrapment syndrome“ (ACNES)	Einmalige Injektion mit Lidocain 1 % als abdomineller Faszienblock, Bericht einer unmittelbaren > 50 %igen Schmerzreduktion: Placebo: 16 %, Lidocain: 54 %
	Lidocain (*n* = 24)	
	NaCl 0,9 % (*n* = 24)	
Labat et al. [24]	Randomisierte kontrollierte Studie zur Pudendusneuralgie, *n* = 201, CT-gestützte Infiltration:	Schmerzresponse nach 3 Monaten: 11,8 % der LA-Gruppe, verglichen mit 14,3 % LA-Gruppe mit Kortikoid (nicht signifikant)
	*n* = 68 Lokalanästhetika (LA)	
	*n* = 66 Lokalanästhetika + Kortison	
	*n* = 67 Lokalanästhetika + Kortison + Kochsalz	
Miclescu et al. [26]	Randomisierte kontrollierte Studie im Cross-over-Design bei *n* = 16 Patient:innen mit schmerzhaften Neuromen nach Nerventrauma Lokale Injektion von	Reduktion spontaner Schmerzen durch Lidocaininfiltration am Neurom, unmittelbar nach Injektion deutlich in beiden Gruppen
	Gruppe A: 0,5 % Lidocain	
	Gruppe B: 0,1 % Lidocain	

## Diskussion

Die vorliegende Studie berichtet über die aktuelle Evidenzbewertung zur Anwendung von Lokalanästhetika als Infiltration oder kontinuierliche Applikation bei der peripheren Neuropathie nach einer Nervenverletzung. Insgesamt zeigen sich eine bemerkenswerterweise geringe Anzahl an relevanten Studien und ein folglich limitiertes Level an Evidenz, welches allerdings nunmehr immerhin eine klinische Studie aus dem Jahr 2021 umfasst [21]. Trotz des guten Studiendesigns dieser randomisierten klinischen Studie ist leider kein längerfristiges Therapieziel über 4 Wochen ohne Selektionsbias bewertbar, da durch das Cross-over-Design ein Teil der Studienpopulation die Therapiegruppe wechselte. Die hier beschriebene Praxis der ambulanten, kontinuierlichen Lokalanästhesie ist aktuell in Deutschland nicht etabliert, allerdings in der Literatur bereits beispielsweise aus Bologna in Italien berichtet worden [5]. Des Weiteren sind zur eingeschlossenen Studie inzwischen Folgepublikationen erschienen, in denen sekundäre Endpunkte in der gleichen Population berichtet worden sind [19, 20].

## Weitere Studien

Nur eine Studie erfüllte die engen, a priori festgelegten Ein- und Ausschlusskriterien des systematischen Reviews. Darüber hinaus ist jedoch eine Reihe weiterer Studien erwähnenswert und in Tab. 3 charakterisiert. So zeigte sich in der kritischen Literaturdurchsicht im Rahmen der Volltextanalyse, dass zu spezifischen Indikationen von Engpasssyndromen wie der Pudendusneuralgie [24] oder dem „anterior cutaneous nerve entrapment syndrome“ (ACNES) erfolgreiche Blockaden mit Lokalanästhetika in Studien beschrieben wurden [4]. Auch für den persistierenden Kniegelenkschmerz nach einer Totalendoprothese bei Neuropathie des Ramus infrapatellaris berichtet eine Fallserie über eine sehr hohe Ansprechrate [7]. In diesen Studien hatte die interventionelle Lokalanästhetikainfiltration zunächst einen diagnostischen Stellenwert, konnte jedoch auch eine nachhaltige Schmerzreduktion erreichen. Einige Autoren berichten zu speziellen Indikationen nach individueller Nutzen-Risiko-Abwägung auch eine wiederholte Infiltration mittels Lokalanästhetika [13]. Zusätzlich zu erwähnen wären zudem die Empfehlungen der International Association for the Study of Pain Neuropathic Pain Special Interest Group (NeuPSIG), die invasive Verfahren bei sonst therapierefraktären Schmerzen als therapeutische Option erwähnen [10]. Der pathophysiologische Mechanismus von Schmerzen nach Extremitätenamputation beinhaltet verschiedene neuronale Aspekte und stellt aufgrund der hohen Prävalenz von Phantomschmerz nach Amputationen weiterhin eine schmerztherapeutische Herausforderung dar [33]. Pathophysiologisch werden supraspinale, spinale Reorganisationsprozesse und periphere Mechanismen u. a. mit Inflammationsprozessen diskutiert [18]. In diesem Kontext erwähnenswert ist die Studie von Haroutounian et al., in der bei neuropathischen Schmerzen die Rolle des peripheren Input untersucht wurde. Während die systemische Gabe von Lidocain als Infusion zu einer Schmerzreduktion führte, war bei perineuraler Gabe eine umgehende Schmerzfreiheit zu erreichen und eine quantitative sensorische Testung erfolgt. Die Autoren schlussfolgern, dass periphere Afferenzen die spontane Schmerzintensität unterhalten und nicht ausschließlich zentrale Effekte eine Rolle spielen können [17]. Ein Erklärungsmodell für diese Beobachtung haben Gangadharan und Kuner et al. kürzlich in experimentellen Publikationen erbracht [12].

Die vorliegende Arbeit berichtet über den aktuellen Stand der Literatur zu einer wiederkehrenden Fragestellung minimalinvasiver Therapieangebote in der Schmerztherapie. Trotz der langen Tradition lokalanästhesiologischer Behandlungsstrategien, den exzellenten Erfahrungen von Anästhesist:innen nicht zuletzt durch Entwicklungen der sonographiegesteuerten Lokalanästhesie und der Häufigkeit des Krankheitsbildes waren überraschend wenig konfirmatorische Studien zu identifizieren. Die Suchstrategie folgte den Standards von S3-Leitlinien und umfasste die gängigen Datenbanken. Insbesondere ältere Daten, die nicht in einer klinischen Studie oder in Buchform publiziert worden sind, können jedoch einer entsprechenden Suche entgehen. Eine quantitative Datenanalyse konnte hier nicht durchgeführt werden und die Literaturanalyse muss sich auf eine qualitative Zusammenfassung beschränken. Nicht von der Hand zu weisen, bleibt die Frage eines Publikationsbias, da auch im Rahmen der Literaturanalyse publizierte Studienprotokolle identifiziert wurden. So ist eine Untersuchung trotz formellem Studienabschluss in 2018 bislang nicht mit Studienergebnissen publiziert, und mehrere Anfragen an die Autoren blieben ohne Antwort [3]. Schlussendlich kommt der Prävention von Phantomschmerzen aufgrund der komplexen Therapie eine besondere Rolle zu [33]. Ein therapeutisches Angebot in entsprechend gelagerten Fällen wäre insofern aus der gesichteten Literatur ableitbar. 

### Infobox Suchstrategie in PubMed in der Literaturanalyse

(„Peripheral Nerve Injuries“[MeSH Terms] OR ((„peripheral nerves“[MeSH Terms] OR „Peripheral Nervous System“[MeSH Terms] OR („peripheral“[TIAB] AND „nerves“[TIAB]) OR „peripheral nerves“[TIAB] OR („peripheral“[TIAB] AND „nerve“[TIAB]) OR „peripheral nerve“[TIAB] OR „nerve“[TIAB] OR „nervus “[TIAB]) AND („injuries“[MeSH Subheading] OR „injur*“[TIAB] OR „trauma*“[TIAB] OR „wounds and injuries“[MeSH Terms] OR („wounds“[TIAB] AND „injuries“[TIAB]) OR „wounds and injuries“[TIAB] OR „traumas“[TIAB] OR „traumas“[TIAB] OR „accident*“[TIAB] OR „iatrogen*“[TIAB]))) AND (english[Filter] OR german[Filter] OR French[Filter]) NOT (((„animals“[MeSH Terms] OR „animal*“[TIAB] OR „rat“[TIAB] OR „rats“[TIAB] OR „mouse“[TIAB] OR „mice*“[TIAB]) NOT „humans“[MeSH Terms]) OR („Olfactory Nerve Injuries“[MeSH Terms] OR „olfactory nerve*“[TIAB] OR „Optic Nerve Injuries“[MeSH Terms] OR „optic nerve*“[TIAB] OR „Oculomotor Nerve Injuries“[MeSH Terms] OR „Oculomotor nerve*“[TIAB] OR „Abducens Nerve Injury“[MeSH Terms] OR „abducens nerve*“[TIAB] OR „Trochlear Nerve Injuries“[MeSH Terms] OR „trochlear nerve*“[TIAB] OR „Vestibulocochlear Nerve Injuries“[MeSH Terms] OR „vestibulocochlear nerve*“[TIAB]) OR („Case Reports“[PT] OR „case report*“[TIAB])) AND („Neuralgia“[Mesh] OR „neuropathic*“[TIAB] OR „neuropathic pain*“[TIAB] OR „nerve pain*“[TIAB] OR „Phantom Limb“[Mesh] OR „phantom pain*“[TIAB] OR „phantom pain*“[TIAB] OR („phantom pain*“[TIAB] AND „Limb*“[TIAB]) OR „Pseudomelia“[TIAB]) AND (Randomized Controlled Trial[PT] OR Controlled Clinical Trial[PT] OR Pragmatic Clinical Trial[PT] OR Randomized[TIAB] OR Randomised[TIAB] OR Placebo[TIAB] OR Randomly[TIAB] OR „Controlled“[TIAB] OR „Control group“[TIAB] OR (Intervention[TIAB] AND Control[TIAB])).

## Fazit für die Praxis

- Invasive Verfahren können eine diagnostische Maßnahme sein, um den Einfluss von peripherem sensorischem Input und die Schmerzlokalisation zu sichern.
- Als therapeutische Intervention kann eine perineurale Lokalanästhetikainfiltration durchgeführt werden. Eine schwache Empfehlung für die Intervention ist neu in die Leitlinie aufgenommen worden.
- Zu Zeitdauer der Wirksamkeit und Frequenz von therapeutischen Blockaden liegen aktuell keine Studien vor, um ein spezifisches Vorgehen abzuleiten.

## Data Availability

Die in dieser Studie erhobenen Datensätze können auf begründete Anfrage beim Korrespondenzautor angefordert werden.
